# Structure‐Activity Relationship and Crystallographic Studies on 4‐Hydroxypyrimidine HIF Prolyl Hydroxylase Domain Inhibitors

**DOI:** 10.1002/cmdc.201900557

**Published:** 2019-12-03

**Authors:** James P. Holt‐Martyn, Rasheduzzaman Chowdhury, Anthony Tumber, Tzu‐Lan Yeh, Martine I. Abboud, Kerstin Lippl, Christopher T. Lohans, Gareth W. Langley, William Figg, Michael A. McDonough, Christopher W. Pugh, Peter J. Ratcliffe, Christopher J. Schofield

**Affiliations:** ^1^ Department of Chemistry University of Oxford Chemistry Research Laboratory 12 Mansfield Road Oxford OX1 3TA UK; ^2^ NDM Research Building University of Oxford Oxford OX3 7FZ UK; ^3^ The Francis Crick Institute London NW1 1AT UK

**Keywords:** anaemia, hypoxia, prolyl hydroxylases, structure-activity relationships

## Abstract

The 2‐oxoglutarate‐dependent hypoxia inducible factor prolyl hydroxylases (PHDs) are targets for treatment of a variety of diseases including anaemia. One PHD inhibitor is approved for use for the treatment of renal anaemia and others are in late stage clinical trials. The number of reported templates for PHD inhibition is limited. We report structure–activity relationship and crystallographic studies on a promising class of 4‐hydroxypyrimidine‐containing PHD inhibitors.

## Graphical abstract

**PHD candidates**: The 2‐oxoglutarate‐dependent hypoxia inducible factor prolyl hydroxylases (PHDs) are targets for treatment of a variety of diseases including anaemia. One PHD inhibitor is approved for use for the treatment of renal anaemia and others are in late‐stage clinical trials. However, the number of reported templates for PHD inhibition is limited. We report structure‐activity relationship and crystallographic studies on a promising class of 4‐hydroxypyrimidine‐containing PHD inhibitors.

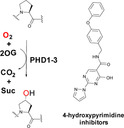

Inhibition of the hypoxia inducible factor (HIF) prolyl hydroxylases (human PHD 1–3), with consequent increases in HIF levels, is being pursued for treatment of anaemia (*via* increasing erythropoietin) and has potential for treatment of other ischemia‐related diseases.^1^ The PHDs are Fe(II)/2‐oxoglutarate (2OG) oxygenases that catalyse hydroxylation of prolyl‐residues in the oxygen degradation domains (ODDs) of HIFα (Figure 1A).^2^ The oxygen‐dependent prolyl hydroxylation of HIFα isoforms signals for their degradation *via* the ubiquitin‐proteasome system. As oxygen levels decrease, HIF‐α levels rise and HIFα dimerizes with HIFβ. The HIFα,β complex promotes the context‐dependent transcription of specific gene sets.^1^, ^2^ PHD inhibitors are in clinical trials for anaemia treatment in chronic kidney disease, with Roxadustat (**1**) being recently approved for use in dialysis patients in China (Figure 1B).2a–2c, 3 Most PHD inhibitors chelate to the active site Fe(II) and compete with 2OG (*e. g*., **3**, Figure 2A) and, to differing extents, with HIFα.2b, 3a, 4 It is likely none of the current ‘clinical’ PHD inhibitors are completely selective for the PHDs over other human 2OG oxygenases.2b, 5 Since many human 2OG oxygenases are involved in disease/biologically important processes,^6^ there is a need for new scaffolds for PHD, and other 2OG oxygenases, inhibition.

**Figure 1:**
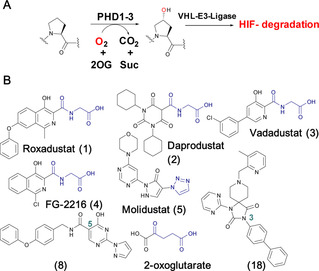
HIF prolyl hydroxylases are therapeutic targets. A. Prolyl‐4‐hydroxylation of hypoxia inducible factor α (HIFα) subunits signals for their degradation via the ubiquitin proteasome system. 2OG, 2‐oxoglutarate; Suc, succinate; PHD1‐3, human prolyl hydroxylase enzymes 1–3; VHL−E3 ligase, the von Hippel‐Lindau protein (VHL) is the targeting component of a ubiquitin E3 ligase system. B. Examples of PHD inhibitors. Roxadustat (FG‐4592, **1**), Daprodustat (GSK1278863, **2**), Vadadustat (**3**), FG‐2216 (**4**) and Molidustat (BAY 85‐3924, **5**). Representative 4‐hydroxypyrimidine (**8**) and spiro[4.5]decanone (**18**) inhibitors are shown, the biaryl unit of the latter binds in a hydrophobic pocket close to the PHD active site.

**Figure 2:**
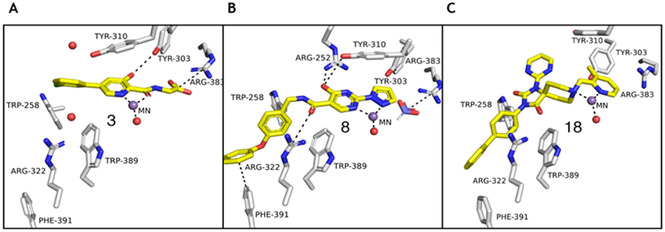
Comparison of views from crystal structures of PHD2.Mn^II^ in complex with (A) **3** (PDB 5OX6),^7^ (B) **8**, and (C) **18** (PDB 6QGV). Analysis of the binding modes of **8** (A, B) reveals that its pyrazole ring occupies the 2OG binding pocket and chelates to the active site metal (Mn substituting for Fe) in a bidentate manner. Note the extent to which the PHD inhibitors project into the substrate binding pocket varies. In the case of the 4‐hydroxypyrimidine inhibitors (B), the biphenyl group projects into a hydrophobic pocket formed by the side chains of Trp‐258, Trp‐389 and Phe‐391, which are involved in substrate binding. The inhibitors also interact with the catalytically important residue Arg‐322.

Recently, we reported SAR studies on spiro[4.5]decanone containing PHD inhibitors, leading to the identification of a hydrophobic pocket close to the Fe(II) binding site of the target enzymes.^7^ The side chains of Trp‐258, Trp‐389 and Phe‐391 in PHD2 are positioned to make hydrophobic and π‐stacking interactions with the biphenyl substituent of spiro[4.5]decanone derivatives.^7^ We now describe SAR and crystallographic studies on potent 4‐hydroxypyrimidine PHD inhibitors, which exploit binding in this hydrophobic pocket.

To explore diversification of binding in the hydrophobic pocket at the PHD active site entrance, we selected the 4‐hydroxy‐2‐(1*H*‐pyrazol‐1‐yl)pyrimidine scaffold because we predicted its *C*‐5 amide group would bind to the PHDs analogously to the C‐3 group of the spiro[4.5]decanone series.^8^ This series is of interest from both clinical application and chemical probe perspectives because it has provided PHD inhibitors that are orally bioavailable in several animal species.^8^ Like Molidustat, but unlike most PHD inhibitors in clinical development, the hydroxypyrimidines do not contain a carboxylic acid, a possible pharmacokinetic advantage from an *in vivo* use perspective. Some SAR studies on the 4‐hydroxy‐2‐(1*H‐*pyrazol‐1‐yl)‐pyrimidine PHD inhibitors are reported,^8^ but no details of their binding modes or selectivity *versus* other 2OG oxygenases have been described.

We initially targeted biphenyl derivatives **8** and **9** for PHD inhibition. A series of other *C*‐5 amide derivatives (**10**–**17**) was subsequently made to explore more detailed SAR (Scheme 1). 4‐Hydroxy‐2‐(1*H*‐pyrazol‐1‐yl)pyrimidine (**6**) was synthesized as reported, *via* cyclization of diethyl‐ethoxy methylene malonate and 1*H*‐pyrazole‐1‐carboximidamide; lithium hydroxide mediated ester hydrolysis of **6** gave **7**.^8^ The reported amide coupling conditions failed to efficiently produce the desired amides. It is reported that the air stable Al adduct (AlMe_3_)_2_DABCO (DABAL‐Me_3_) can be used to form amides directly from methyl esters and amines, suggesting hydrolysis of **6** may not be required for amide formation. Thus, **6** was reacted with DABAL‐Me_3_ then with the relevant amine to yield **8**–**17**.

**Scheme 1:**
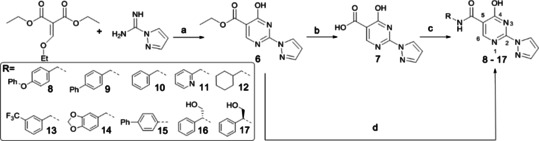
Synthesis of 4‐hydroxypyrimidine series (**8**–**17**) to investigate the role of the C‐5 amide group in PHD inhibition. (a) NaOEt, Ethanol, microwave, 90 °C, 2 hrs. (b) NaOH, THF:H_2_O (10 : 1), rt, 16 hrs. (c) (i) 1,1’‐carbonyldiimidazole (CDI), DMAc, 100 °C, 30 min. (ii) R‐NH_2_, 16 hrs, rt (d) (AlMe_3_)_2_DABCO (DABAL‐Me_3_), R‐NH_2_, THF, microwave, 130 °C, 10 min.

**6**, **8**–**17** were then assayed using a mass spectrometry (MS) based assay for PHD2 inhibition using a human HIF‐1α *C*‐terminal ODD (CODD) fragment.^7^ The results imply the importance of the *C*‐5 amide aryl substituent: **6** was inactive, whereas **8** and **9** were potent inhibitors (IC_50_ **8**, 0.256 μM; **9**, 0.210 μM). **10** and **11** (IC_50_ **10**, 0.396 μM; IC_50_ **11**, 0.950 μM) were not as potent as **8** or **9**, emphasizing the importance of the *C*‐5 amido group. The results for **13**–**17** reveal potential for optimization involving interactions in the hydrophobic pocket. Thus, the 3‐trifluoromethylbenzyl (IC_50_ **13**, 0.153 μM) and benzo[*d*]‐[1,3]dioxole (IC_50_ **14**, 0.261 μM) derivatives achieved similar levels of inhibition to **8**. Removal of the methylene linker as in **15** (IC_50_ 0.213 μM) manifested similar levels of potency to **9**, suggesting a methylene link is not essential for potent inhibition. Addition of a hydroxymethylene group on the methylene linker (IC_50_ **16**, 0.093 μM; IC_50_ **17**, 13.2 μM) reveals the impact of introducing chirality into the scaffold. Thus, (*R*)‐**16** manifested improved (4‐fold) PHD2 inhibition compared to **10**, whereas (*S*)‐**17** was much less active.

An X‐ray crystal structure of a truncated domain of PHD2 (tPHD2) with Mn^II^ (substituting for Fe^II^) and complexed with **8** was obtained (Figure 2B, **S1**–**4**). The overall fold is similar to previously reported PHD‐inhibitor complex structures (backbone root mean square deviation with a structure of tPHD with **18**: 0.7564) (Figure 2C, **S2**‐**3**).^7^ The structure reveals a binding mode for **8** involving chelation of the active site metal *via* nitrogen atoms of the pyrazolo and a pyrimidyl rings which adopt a coplanar conformation (Figure 2B, **S1**–**4**). Octahedral metal ion coordination is completed by monodentate chelation by the conserved 2His‐Asp metal binding triad of the PHDs and a water molecule (**Figure** **S1**–**4**). The pyrazole ring of **8** occupies the entrance of the active site pocket that is occupied by the CH_2_CO_2_H group of 2OG during catalysis. The conformation of the side chain of Arg‐383 which interacts with 2OG C‐5 carboxylate/analogous carboxylate in many PHD inhibitors (*e. g*. **3**, as in Figure 2A) is different in the complex with **8**, likely reflecting the lack of a carboxylate/carboxylate equivalent in **8**. Instead of interacting with an inhibitor carboxylate, the guanidino group of Arg‐383 is positioned to interact with a formate ion, likely derived from the crystallization buffer. The hydroxyl group of the pyrimidine of **8** is positioned to H‐bond with Arg‐252 and Tyr‐310 (**Figure** **S1**).

As proposed, the 4‐phenoxy‐phenyl substituent of **8** is located in the hydrophobic pocket, formed by residues including Trp‐258, Trp‐389 and Phe‐391 (**Figure** **S4**). The addition of an ether link between the phenyl rings of **8** apparently results in a different π‐π interaction compared to the C‐3 biphenyl substituent of **18** (Figure 2B **& 2** **C**).^7^ The terminal phenyl ring of **8** forms an edge‐face π‐stacking interaction with Phe‐391 and interacts with the Arg‐396 side chain (Figure 2B). The Arg‐322 side chain is positioned to interact with **8** in an analogous manner to how it interacts with the spiro[4.5]decanone inhibitors, *i. e*. to form a cation‐π interaction with the NCH_2_‐phenyl ring of **8** and to H‐bond with its amide carbonyl oxygen (Figure 2B).^7^

The extent of PHD2 inhibition was similar when using HIF‐1α *N*‐terminal ODD (NODD) and CODD substrates in terms of rank order of potency, though the IC_50_ values for NODD were generally lower (Table 1).2b, 5 To further investigate their binding with different substrates, **8**, **15**–**17** and **18** (a spiro[4.5]decanone series representative) were tested for CODD and NODD substrate displacement from the appropriate PHD2.Zn(II).2OG substrate complex *via* reported 1D CLIP HSQC NMR analyses (with selective ^13^C‐inversion) (**Figure** **S5**).^9^ Notably, all tested compounds displaced the HIF‐1α NODD, but not the CODD fragment, consistent with the lower IC_50_ values for NODD compared to CODD. Note that binding of the inhibitors may well disrupt CODD binding in the immediate active site (see Yeh *et al*. for discussion).2b

**Table 1:** Studies on 4‐hydroxypyrimidine containing inhibitors. Compounds were screened against PHD2_181‐426_ using HIF‐1α CODD and NODD substrates, FIH using HIF‐1α CAD peptide D788–L822, KDM4A using H3(1–15) K9Me3. PHD2, FIH, and KDM4A assays employed a RapidFire MS machine. Standard errors of the mean are reported (n=3).

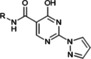					
	R	PHD2 with HIF‐1α CODD IC_50_ μM	PHD2 with HIF‐1α NODD IC_50_ μM	FIH IC_50_ μM	KDM4A IC_50_ μM
*6*	precursor	>25	>25	>25	>25
*8*	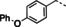	0.256±0.088	0.094±0.042	>25	>25
*9*	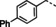	0.210±0.051	0.093±0.033	>25	20
*10*	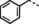	0.396±0.180	0.122±0.065	>25	>25
*11*	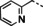	0.950±0.185	0.359±0.110	>25	17
*12*	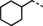	>25	>25	>25	>25
*13*	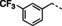	0.153±0.042	0.066±0.026	>25	>25
*14*	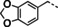	0.261±0.037	0.274±0.034	>25	23
*15*	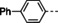	0.213±0.064	0.101±0.023	>25	>25
*16*	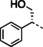	0.093±0.039	0.027±0.012	>25	>25
*17*	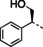	13.275±3.4	3.95±1.62	>25	21

*18*	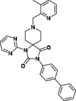	0.253±0.047	0.127±0.056	>25	4.69

**8**–**18** were tested for inhibition of other 2OG oxygenases (Table 1) using solid phase extraction linked to MS (RapidFire MS) or MALDI MS based assays.2a, 2b, 5, 10 **8**–**18** showed little/no inhibition of human FIH (factor inhibiting HIF, a JmjC ‘hydroxylase’) and KDM4A (a JmjC demethylase) using RapidFire MS assays. By contrast, spiro[4.5]decanone **18** was active against KDM4A (IC_50_ **18**, 4.65 μM) (Table 1). Results (using MALDI MS) with two other prolyl hydroxylases, human OGFOD1 and a viral collagen prolyl hydroxylase (vCPH, a model for human collagen type prolyl hydroxylases) (Table 1) reveal the importance of the *C*‐5 amido group identity in obtaining selectivity (**Figure** **S6**).^5^, ^11^ As with PHD2, **6** was inactive *versus* vCPH; **8** and **16** were potent vCPH inhibitors (IC_50_ **8**, 2.0±0.1 μM; IC_50_ **16**, 1.1±0.1 μM), with **15** being less potent (28.2±1 μM). Notably, **18** was inactive *versus* vCPH (IC_50_>100 μM). All compounds manifested poor OGFOD1 inhibition: at 10 μM inhibitor, 100 % activity relative to control was observed with **1**, and 83±13, 75±1, 41±5, 70±3 and 45±2.5 % activities were observed with **8**, **13**, **16**, **17**, and **18**, respectively. Given that the ‘hydrophobic pocket region’ residues in vCPH and OGFOD1 are different to those in the PHDs (vCPH: Trp‐89, Trp‐223 and Glu‐122; OGFOD1: Trp‐236 and Asp‐140; PHD2: Trp‐258, Trp‐389 and Phe‐391) (**Figure** **S6**),4a, 12 these results are consistent with the proposal that modulating binding in this pocket is a means to achieve selectivity.

Compounds **8**, **11**, **13**–**18** were tested for cellular activity by measuring cellular HIF‐1α stabilization by immunoblotting with a Hep‐3B human cell line (**Figure** **S7**).2b At 100 μM, most of the tested inhibitors stabilized HIF‐1α levels, with **8**, **15** and **18** (a spiro compound) having the strongest activity (**Figure** **S7**). At 20 μM, only **15** and **18** manifested HIF‐1α stabilization. Notably, (*S)‐* **16** was inactive in cells at 100 μM; by contrast, (*R)*‐**17** showed modest HIF‐1α stabilization, suggesting the different levels of isolated PHD2 inhibition, in part, translate to cellular observations. Other factors including cell penetration and metabolism likely impact on cellular activity.

The results support the potential of the 4‐hydroxy‐2‐(1*H*‐pyrazol‐1‐yl)pyrimidines as potent and selective PHD inhibitors. Targeting the hydrophobic pocket at the entrance to the active site of the PHDs enabled identification of 4‐hydroxy‐2‐(1*H*‐pyrazol‐1‐yl) pyrimidines selective for the PHDs over structurally related 2OG oxygenases. Modulation of inhibitors elements binding in this region will likely be useful for further optimizing 4‐hydroxy‐2‐(1*H*‐pyrazol‐1‐yl)pyrimidines potency, and for improving the cellular activity of the compounds reported here. SAR probing this region will also be applicable to optimizing other PHD (or other human prolyl‐hydroxylases) inhibitor series, including with respect to selectivity over other human 2OG oxygenases.

## Conflict of interest

C.J.S, P.J.R & C.W.P. are co‐founders of a company, ReOx, which aims to exploit basic science discoveries about the hypoxic response for therapeutic benefit.

## Supporting information

As a service to our authors and readers, this journal provides supporting information supplied by the authors. Such materials are peer reviewed and may be re‐organized for online delivery, but are not copy‐edited or typeset. Technical support issues arising from supporting information (other than missing files) should be addressed to the authors.

Supplementary
