# Supplementary material for: Structure‐Activity Relationship and Crystallographic Studies on 4‐Hydroxypyrimidine HIF Prolyl Hydroxylase Domain Inhibitors
Source: ChemMedChem. 2019 Dec 3;15(3):270–3. doi: 10.1002/cmdc.201900557 (PMC7496690; doi:10.1002/cmdc.201900557)
Supplement: Supplementary file 1 — Supplementary [file CMDC-15-270-s001.pdf]

CHEMMEDCHEM

Supporting Information

Structure-Activity Relationship and Crystallographic Studies on 4-Hydroxypyrimidine HIF Prolyl Hydroxylase Domain Inhibitors

James P. Holt-Martyn, Rasheduzzaman Chowdhury, Anthony Tumber, Tzu-Lan Yeh, Martine I. Abboud, Kerstin Lippl, Christopher T. Lohans, Gareth W. Langley, William Figg, Jr., Michael A. McDonough, Christopher W. Pugh, Peter J. Ratcliffe, and Christopher J. Schofield*

Contents

Figure S1 ... 4
Figure S2 ... 4
Figure S3 ... 5
Figure S4 ... 6
Figure S5 ... 7
Figure S6 ... 8
Figure S7 ... 9
Data Collection ... 10
General Experimental Methods ... 11
Preparation of tPHD2 (residues 181 - 426) ... 11
X-ray Crystallography ... 11
RapidFire-MS PHD2 hydroxylation assays[1] ... 12
RapidFire-MS KDM4A hydroxylation assays ... 14
MALDI-TOF MS OGFOD1 Hydroxylation Assays ... 15
MALDI-TOF MS vCPH Hydroxylation Assays ... 16
NMR Experiments ... 16
General Considerations For Synthesis ... 17
General Procedure for ethyl ester amide coupling ... 18
Ethyl 4-hydroxy-2-(1H-pyrazol-1-yl) pyrimidine-5-carboxylate 6 ... 18
4-Hydroxy-2-(1H-pyrazol-1-yl)pyrimidine-5-carboxylic acid 7 ... 19
4-Hydroxy-*N*-(4-phenoxybenzyl)-2-(1H-pyrazol-1-yl)pyrimidine-5-carboxamide 8 ... 20
N-([1,1’-Biphenyl]-4-ylmethyl)-4-hydroxy-2-(1H-pyrazol-1-yl)pyrimidine-5-carboxamide 9 ... 20
N-Benzyl-4-Hydroxy-2-(1H-pyrazol-1-yl)pyrimidine-5-carboxamide 10 ... 21
4-Hydroxy-2-(1H-pyrazol-1-yl)-N-(pyridin-2-ylmethyl)pyrimidine-5-carboxamide 11 ... 21
N-(Cyclohexylmethyl)-4-hydroxy-2-(1H-pyrazol-1-yl)pyrimidine-5-carboxamide 12 ... 22
4-Hydroxy-2-(1H-pyrazol-1-yl)-N-(3-(trifluoromethyl)benzyl)pyrimidine-5-carboxamide 13 ... 23
N-(Benzo[d][1,3]dioxol-5-ylmethyl)-4-hydroxy-2-(1H-pyrazol-1-yl)pyrimidine-5-carboxamide 14 ... 23
N-([1,1’-Biphenyl]-4-yl)-4-hydroxy-2-(1H-pyrazol-1-yl)pyrimidine-5-carboxamide 15 ... 24

(*R*)-4-Hydroxy-N-(2-hydroxy-1-phenylethyl)-2-(1H-pyrazol-1-yl)pyrimidine-5-carboxamide 16 24

(*S*)-4-Hydroxy-N-(2-hydroxy-1-phenylethyl)-2-(1H-pyrazol-1-yl)pyrimidine-5-carboxamide 17 25

References 26

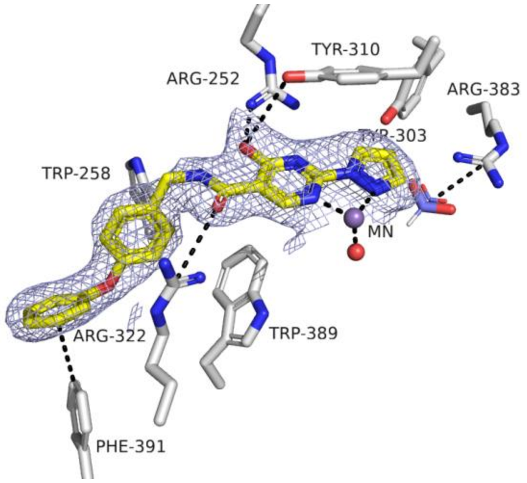

Figure S1: Stereo-view representation of the PHD2.Mn.**8** complex active site showing the OMIT Fo-Fc map (contoured to 1.0 sigma) around the ligand.

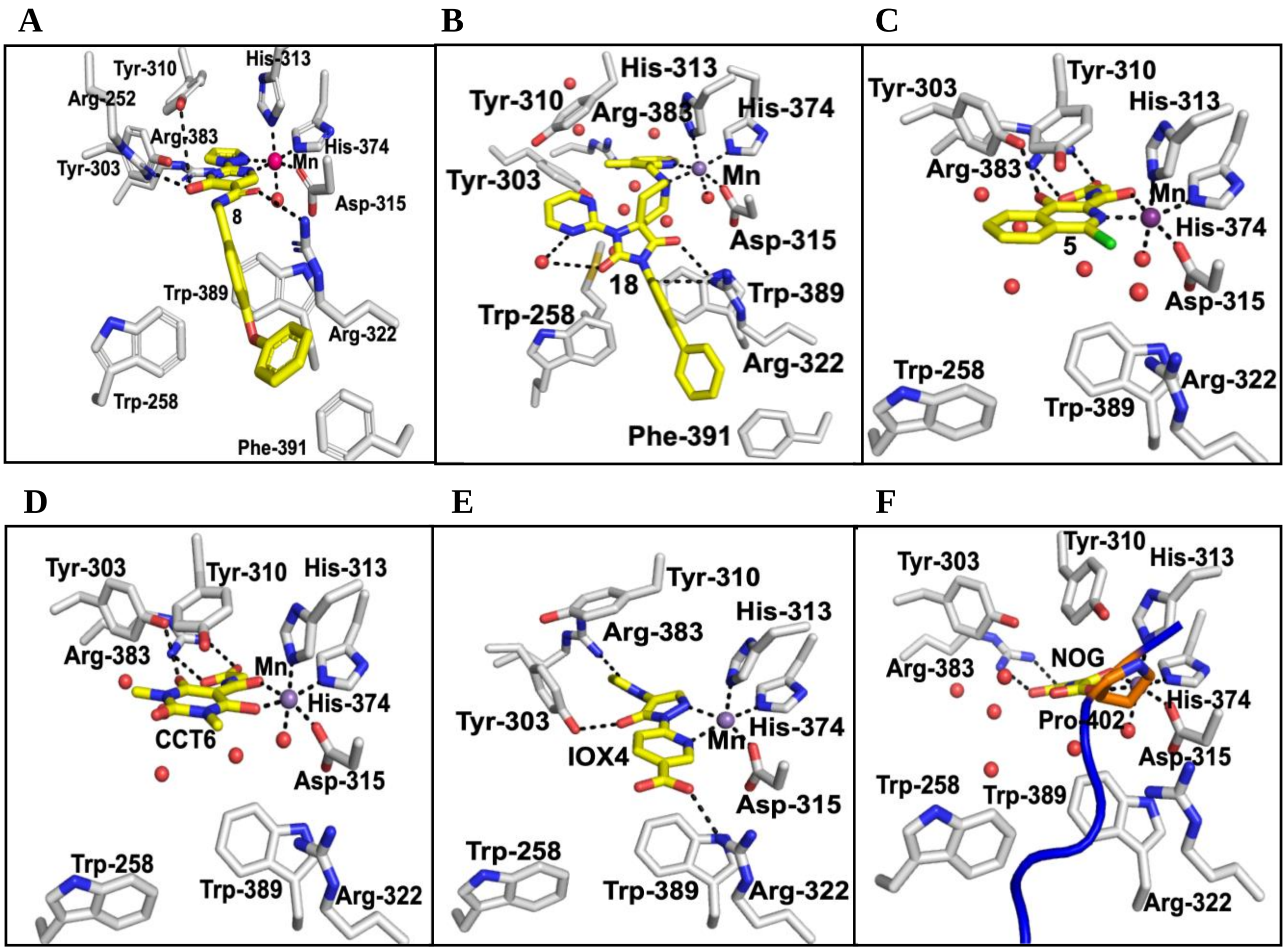

Figure S2: Comparison of views from crystal structures of PHD2.Mn in complex with (**A**) **8**, (**B**) **18** (PDB 6QGV)[1], (**C**) **4** (PDB 5OX6),[4] (**D**) **CCT6** (PDB 5OX5),[2] (**E**) **IOX4** (PDB 5A3U),[3] and (**F**) NOG and the HIF-1α NODD substrate (PDB 5L9V) (Mn substitutes for Fe).[4]

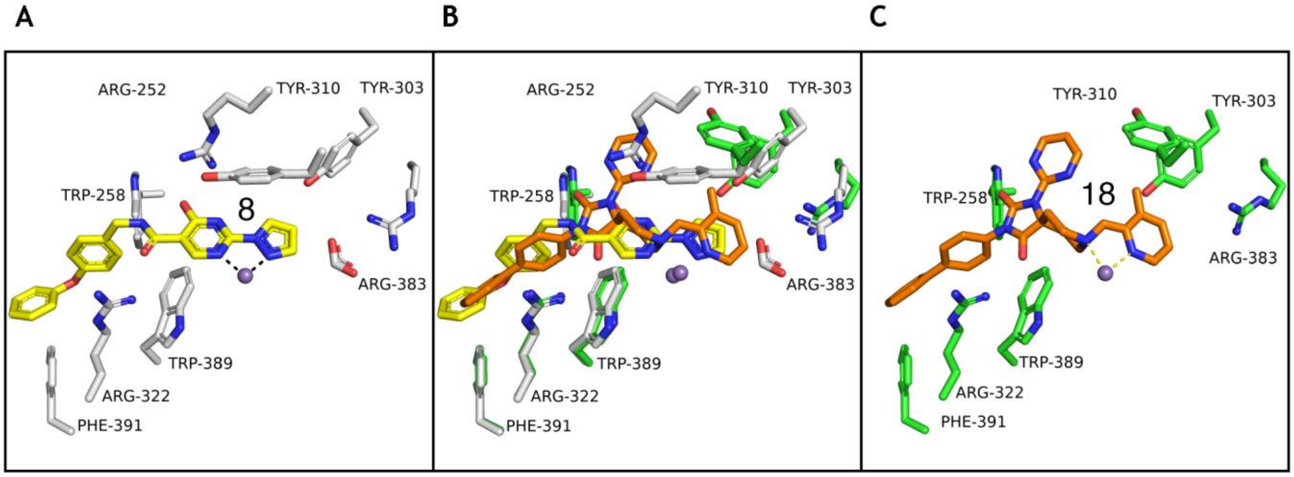

Figure S3: Comparison of views from crystal structures of PHD2.Mn in complex with (A) **8** (PDB ID: 6ST3), overlay of **8** and **18**, and (C) **18** (PDB ID: 6GQV). Note that certain key binding interactions differ between **8** and **18**. **PHD2** Arg-252 is positioned to form a hydrogen-bond interaction with the pyrimidyl alcohol of **8**, whereas in the complex with **18** (C) Arg-252 is apparently disordered. Tyr-310 is positioned to form hydrogen-bond and π-π stacking interactions with the 4-hydroxy pyrimidine of **8** (A), whereas with **18** (C) Tyr-310 adopts a new position to form a hydrophobic / π-π stacking interaction with the 3-methyl pyridine substituent.

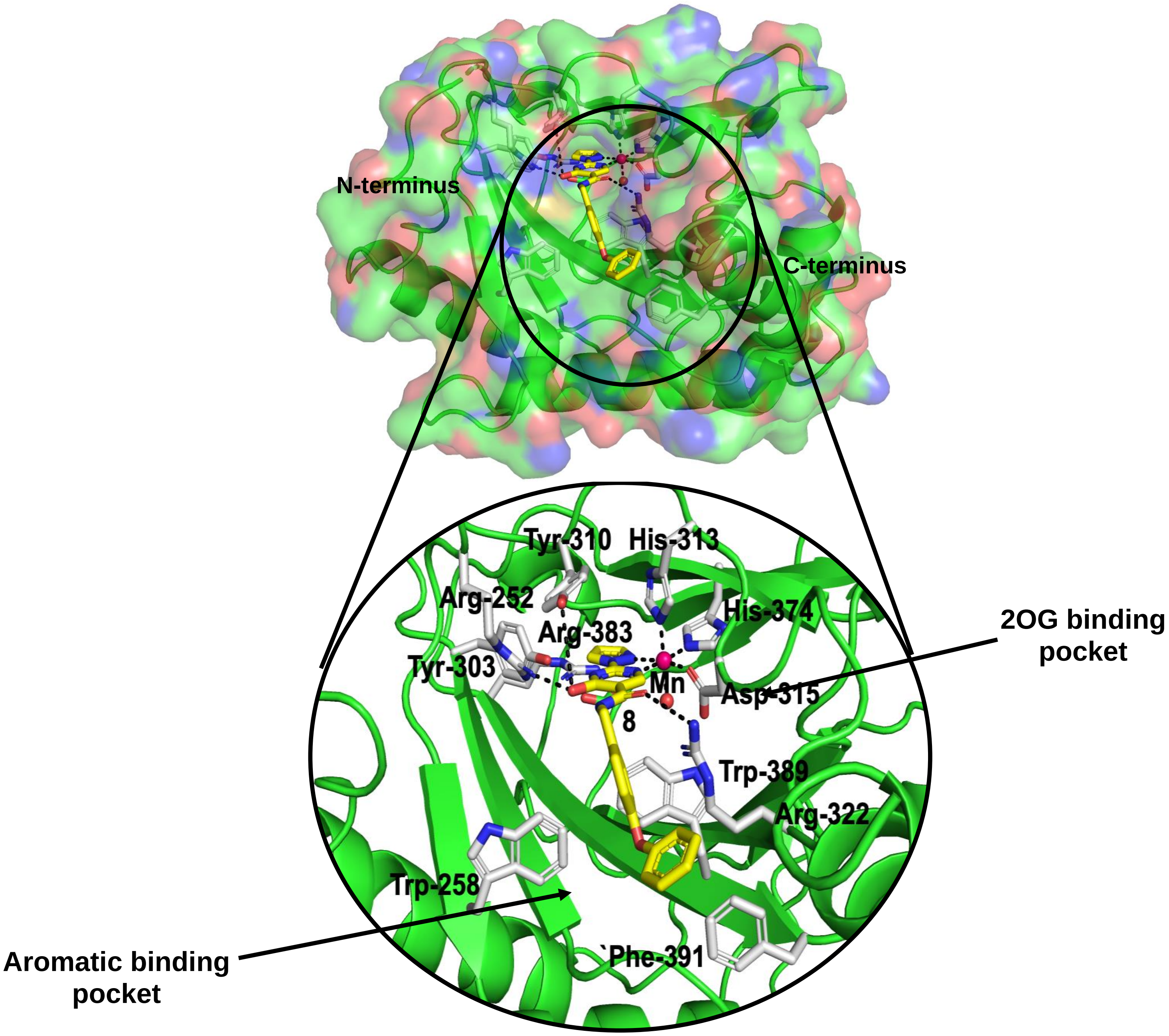

Figure S4: Surface representation view from a crystal structure of PHD2.Mn in complex with **8**. Note that **8** occupies both the 2OG binding pocket and a hydrophobic pocket formed by the side chains of Trp-258, Trp-389 and Phe-391.

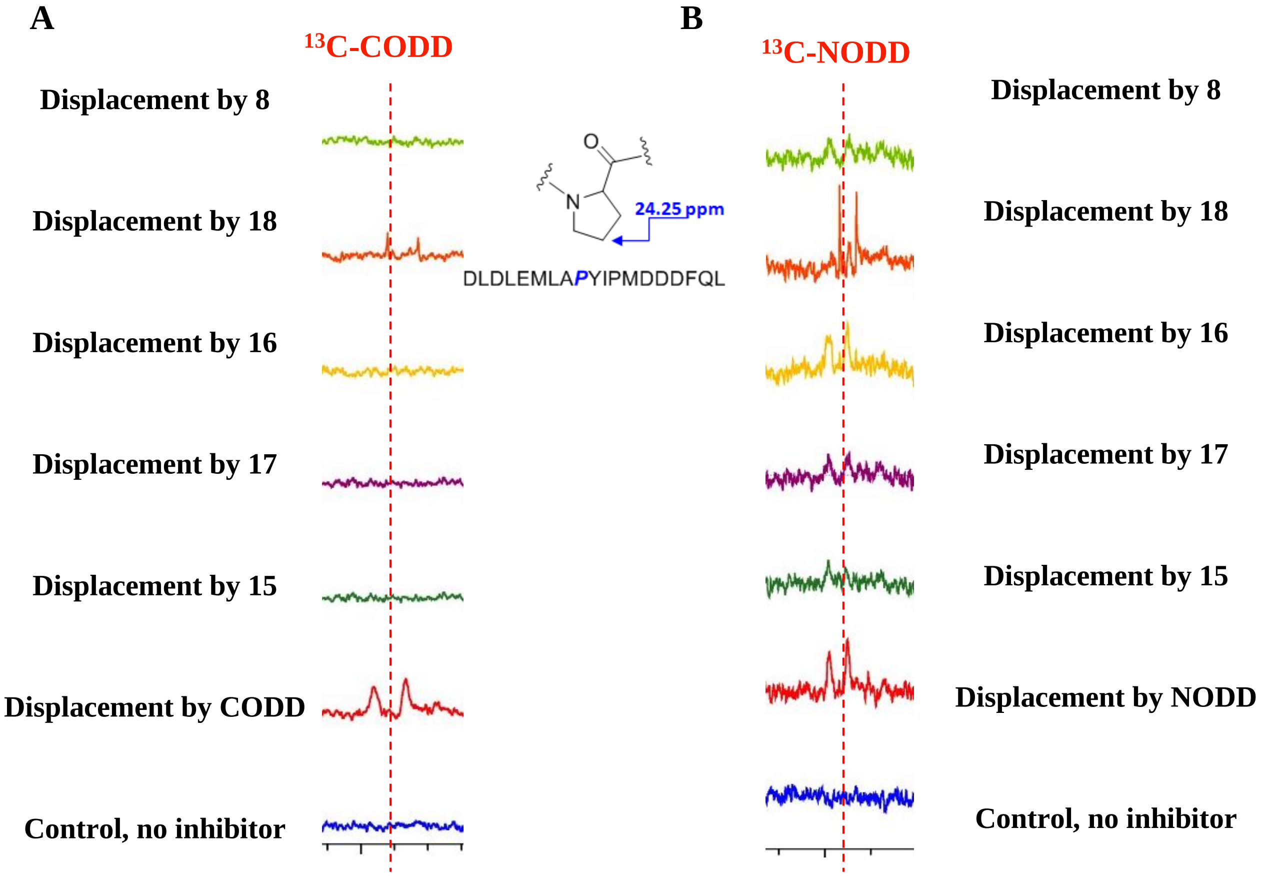

Figure S5: (**A**) Qualitative single concentration screening with various compounds by ^{13}C-CODD displacement as monitored by 1D CLIP HSQC with selective ^{13}C-inversion analyses. (**B**) Qualitative single concentration screening with various compounds by ^{13}C-NODD displacement as monitored by 1D CLIP HSQC with selective ^{13}C-inversion analyses. The assay mixture contained 50 µM *apo*-PHD2$_{181-426}$, 200 µM Zn(II), 50 µM 2OG, 50 µM ^{13}C-CODD or ^{13}C-NODD, 400 µM compound (where necessary) in 50 mM Tris-D$_{11}$, pH 7.5 in 10 % D_2O and 90 % H_2O.

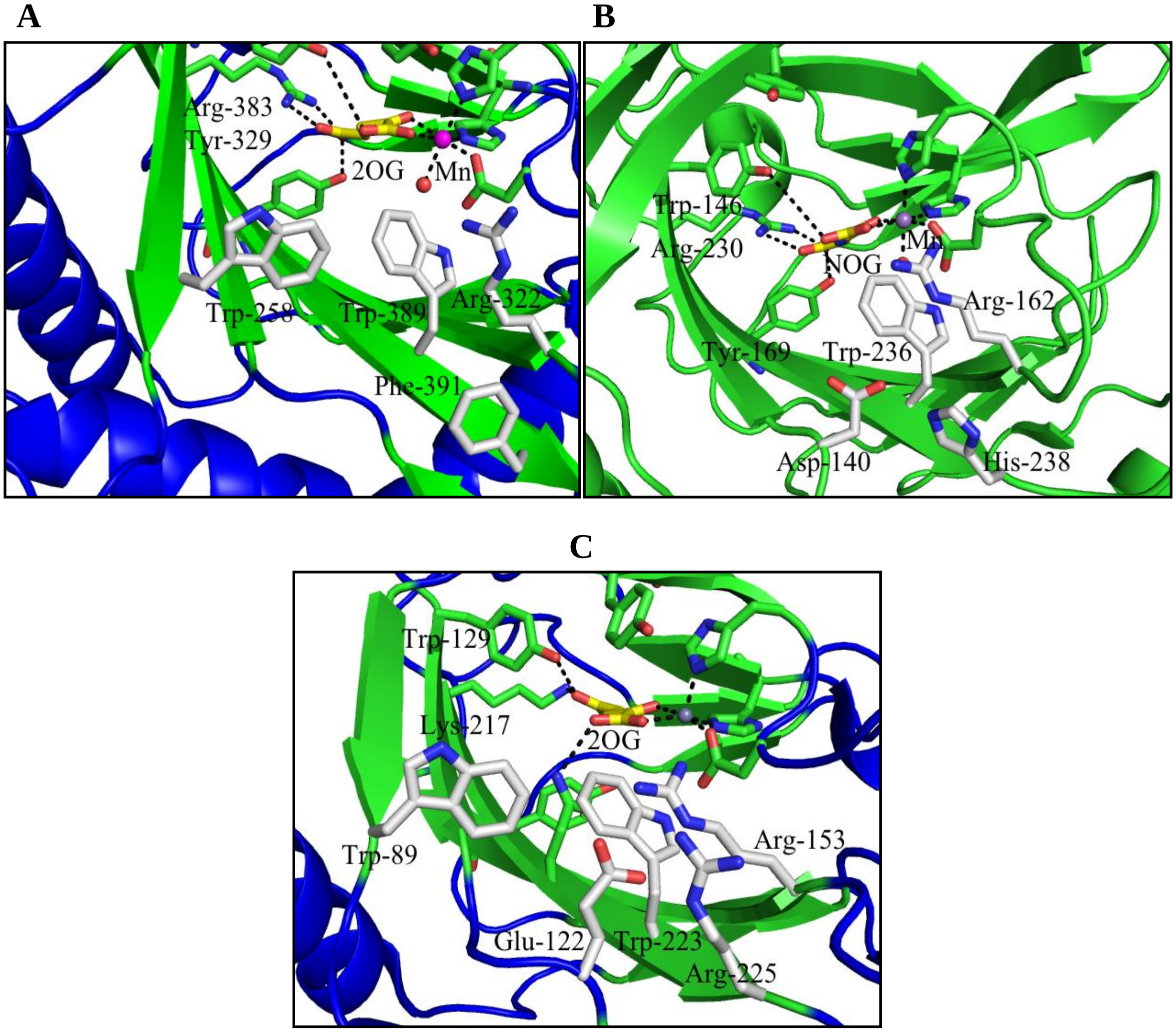

Figure S6: Comparison of active site views from crystal structures of (**A**) PHD2 (PDB ID: 3OUJ)[5], (**B**) OGFOD1 (PDB ID: 4NHX)[6] and (**C**) vCPH (PDB ID: 5C5T)[7]. 2OG, or the 2OG near isostere (*N*-oxalylglycine, NOG) (yellow sticks), bind to a well-defined pocket and chelates the metal in a bidentate manner (Mn substituting for Fe for crystallization purposes). Residues in white sticks include those forming the hydrophobic pocket at the entrance to the active site; other active site residues are in green sticks.

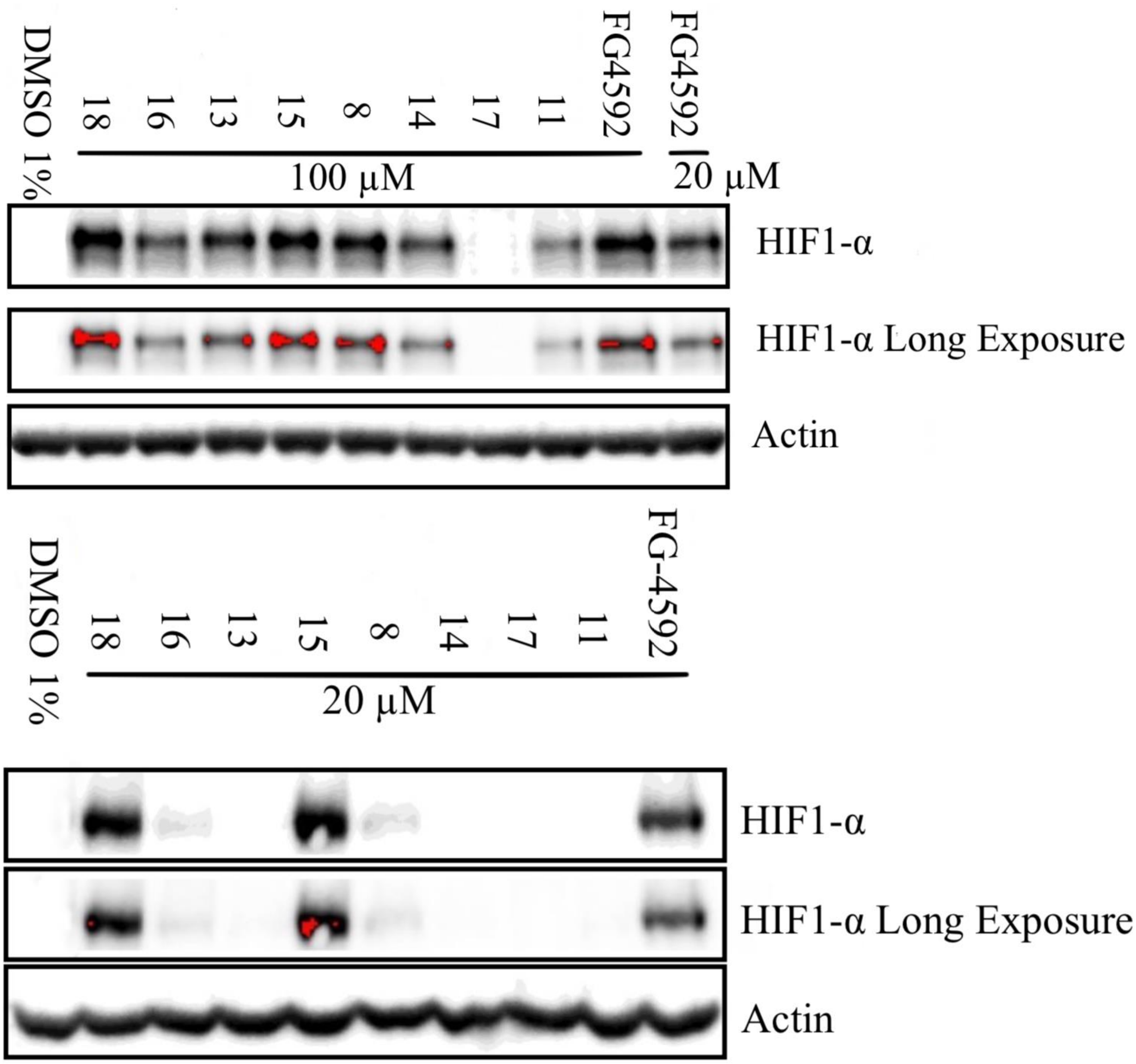

Figure S7: Immunoblots of Hep3B cells treated with 100 μM (**A**) and 20 μM (**B**) PHD inhibitors for 3 hours. The blots show protein levels of HIF1-α and β-actin at the 3rd hour. Protocols for cell culture and immunoblotting have been previously reported.[2]

**PDB acquisition code**	6ST3
**Data Collection**	
Beamline (Wavelength, Å)	0.97625
Detector	Dectris Pilatus 6M-F
Data processing	MOSFLM, SCALA
Space Group	$P2_12 2_1$
Cell dimensions a,b,c (Å)	72.87, 80.58, 83.92
α, β, γ (°)	90, 90, 90
No. of molecules/ ASU	2
Resolution (Å)	83.92-2.43 (2.52-2.43)*
No. of unique reflections	19127 (1978)*
Completeness (%)	99.3 (99.5)*
Redundancy	3.8 (3.8)*
R_{sym}**	0.144 (0.974)*
Mean I/σ(I)	6.9 (1.5)*
Wilson *B* value (Å^2)	35.8
**Refinement statistics**	
R_{factor}‡	0.192
R_{free}‡	0.217
R.m.s. deviation	
Bond length, Å	0.006
Bond angle, °	0.905
**Ramachandran plot (%)**	
Most favored region	98.35
Additionally allowed region	1.65
Disallowed region	0.00

*Highest resolution shell shown in parenthesis.

**$R_{sym} = \Sigma |I-\langle I\rangle| / \Sigma I$, where *I* is the intensity of an individual measurement and $\langle I\rangle$ is the average intensity from multiple observations.

‡$R_{factor} = \Sigma_{hkl}||F_{obs}(hkl)| - k\, |F_{calc}(hkl)|| / \Sigma_{hkl}|F_{obs}(hkl)|$ for the working set of reflections; R_{free} is the R_{factor} for ~5% of the reflections excluded from refinement.

General Experimental Methods

Preparation of tPHD2 (residues 181 - 426)

In brief, cDNA encoding for the catalytic domain of tPHD2 (181-426 residues) was cloned into the pET28a(+)/ pET24a(+) vectors (Novagen), as reported,[8] to enable production of tPHD2 (residues 181-426) protein with/without an N-terminal $His_{6\text{-tag}}$. The tPHD2 (residues 181-426) encoding construct was transformed into the *Escherichia coli* BL21 DE3 cell line; protein production was induced with 0.5 mM isopropyl-b- D-thiogalactosidase (3–5 hr at 28℃). Cells were harvested and lysed by sonication in 20 mM Tris-HCl (pH 7.0) and 0.3 M NaCl; soluble protein (about 5% total soluble extract) was purified by immobilized Ni ion affinity chromatography using pentadentate tris-carboxymethyl ethylene diamine resin followed by cleavage of the His6-tag by thrombin (or alternately by cation exchange chromatography) with a final purification by gel filtration chromatography.[8] The protein was exchanged into 50 mM Tris-HCl buffer (pH 7.5) and concentrated to % 40 mg/ml. The protein was of > 95% purity, as determined by SDS-PAGE analysis and electrospray ionization mass spectrometric analysis.

X-ray Crystallography

Crystallisation procedures were as reported.[1-2, 9] Crystals of truncated PHD2 in complex with **8** were grown in 0.2 M ammonium formate, 0.1 M sodium citrate tribasic dihydrate pH 5.6, 30% PEG 4000 and were cryoprotected by transferring to a solution of mother liquor supplemented with 25% (v/v) glycerol. Data were collected at 100K using synchrotron radiation at the Diamond Light Source (DLS) beamline I03 and were processed by using MOSFLM and SCALA.[10], [11] The structure was solved by molecular replacement using PHASER (search model PDB ID 4BQX)[12] and refined by PHENIX

using the maximum-likelihood function.[13], [14] Iterative cycles of model building in COOT and refinement proceeded until the R_{cryst}/R_{free} values converged.[15] Studies on the optimization of PHD-ligand crystallization conditions will be reported in due course.

RapidFire-MS PHD2 hydroxylation assays[1]

Inhibition of PHD2 activity was assessed by mass spectrometry. The tPHD2 (residues181 - 426) assay monitors turnover of a *C*-terminal oxygenase dependent domain peptide substrate (CODD) DLDLEMLAPYIPMDDDFQL (with a *C*-terminal amide) and appearance of the hydroxylated peptide product (hydroxylation of proline 564) with a typical incubation time of 15 minutes.[2] Assays were performed in 50 mM Tris-HCl pH 7.8, 50 mM NaCl, titrations of compounds for IC_{50} determinations (3-fold and 11-point IC_{50} curves) were performed using an ECHO 550 acoustic dispenser (Labcyte) and dry dispensed into 384-well polypropylene assay plates. The final assay concentration of DMSO was kept constant at 0.5% (v/v). tPHD2 (residues 181 - 426) was used at a concentration of 300 nM in the assay buffer; 25 μL of the tPHD2 solution was dispensed across each 384-well assay plate. tPHD2 (residues 181 - 426) was allowed to equilibrate with compounds for 15 minutes and the enzyme reaction then initiated by dispensing of 25 μL of substrate (20 μM ferrous iron sulfate, 200 μM L-ascrobic Acid, 10 μM CODD or NODD peptide and 20 μM 2OG in the assay buffer).[2] Enzyme reactions were allowed to proceed for 15 minutes and the reaction terminated by addition of 10% (v/v) aqueous formic acid (5 μL). Assay plates were then transferred to a RapidFire RF360 sampling robot (Agilent) connected to an Agilent 6530 quadrupole-time-of-flight (Q-TOF) mass spectrometer. Assay samples were aspirated under vacuum and loaded onto a *C*4 solid phase extraction (SPE) cartridge. After loading the C4 SPE cartridge was washed with 0.1 % formic acid in water to remove non-volatile buffer salts. The peptide was then eluted from the C4 solid phase extraction (SPE) cartridge with 85% acetonitrile, 15% water containing 0.1% formic acid into the mass spectrometer. Peptide charge states were monitored in the positive ion mode. Ion

chromatogram data were extracted for the +2 charge state and peak area data integrated using RapidFire Integrator software (Agilent). The % conversion of the CODD peptide substrate to the +16 hydroxylated peptide was calculated using the equation:

% conversion = 100 x hydroxylated / (hydroxylated + non-hydroxylated peptide) IC_{50} data were determined from non-linear regression plots using GraphPad prism 6.0. The level of +16 (methionine residue oxidation) as observed in the no enzyme control was around 4 - 5%. All data was normalized to no enzyme control.[2]

RapidFire-MS FIH hydroxylation assays

Tris(hydroxymethyl)aminomethane was from Fisher; all other reagents were from Sigma Aldrich and of the highest available purity. Ferrous ammonium sulphate (FAS) was prepared freshly each day as a 400 mM stock solution in 20 mM HCl, this was then diluted to 1 mM in deionized water.[16] L-ascorbic acid (50 mM) and 2OG stock solutions (10 mM) were prepared freshly every day in deionized water, as for the PHD2 assays. The synthetic consensus ankyrin peptide (HLEVVKLLLEAGADVNAQDK-$CONH_2$) was synthesized by GL Biochem (Shanghai) Ltd and dissolved to 1 mM in deionized water.[16-17] FIH was prepared as reported.[2, 18] 20 µL FIH (100 nM) in the assay buffer (50 mM Tris-HCl pH 7.8, 50 mM NaCl) was pre- incubated for 15 minutes in the presence of the inhibitors and the enzyme reaction initiated by addition of 20 µL substrate (200 µM L-ascorbic acid, 20 µM Fe(II) sulfate, 10 µM synthetic ankyrin peptide and 20 µM 2OG).[16] After 15 minutes the reaction was quenched by addition of 4 µL 10% formic acid and the reaction transferred to a RapidFire RF360 high throughput sampling robot. Samples were aspirated under vacuum onto a C4 SPE cartridge. After an aqueous washing step (0.1% (v/v) aqueous formic acid), to remove non-volatile buffer components from the C4 SPE cartridge , peptide was eluted in an organic washing step (85% acetonitrile in water, 0.1% formic acid) and injected into an Agilent 6530 Q-TOF mass spectrometer. Ion chromatogram data was extracted for the non-hydroxylated

peptide substrate and the hydroxylated peptide product; the peak area data for extracted ion chromatograms were integrated using RapidFire Integrator software. % conversion of substrate to product was calculated in Microsoft excel and IC_{50} curves generated using Graph pad Prism.

RapidFire-MS KDM4A hydroxylation assays

The potency of 4-hydroxypyrimidines for KDM4A was assessed by RapidFire mass spectrometry (RF-MS). Truncated KDM4A was prepared as reported.[19] The KDM4A RF-MS assay measures depletion of a histone H3-K9 (Me3) peptide substrate and appearance of the dimethyl peptide product. 2-(N-Morpholino) ethanesulfonic acid (MES) buffer was from Sigma Aldrich.[20] L-Ascorbic acid (LAA, code 11140), ferrous ammonium sulphate (FAS, code 215406) and 2-oxoglutarate (2OG, code K3752) were from Sigma Aldrich (Poole, Dorset). The H3K9Me3 peptide was synthesized by Peptide Protein Research (Fareham, Hampshire).[20] LAA was prepared freshly each day to a concentration of 50 mM in deionized water, ferrous ammonium sulphate was prepared freshly each day by dissolving to 400 mM in 10 mM HCl and then dilution to 1 mM in deionized water, 2OG was prepared freshly each day by dissolving in 10 mM in deionized water. The KDM4A RF-MS assay was performed in 384-well plate format in assay buffer (50 mM MES pH 7.0). All reagent dispenses were performed using a multidrop reagent dispenser (Thermo). Dilutions of compounds were prepared using an ECHO 550 acoustic dispenser (Labcyte) and dry dispensed (250 nL) in duplicate into 384-well polypropylene plates (Greiner Bio-One); DMSO (250 nL) was dispensed into columns 12 and 2, 4-pyridinedicarboxylic acid (a known JmjC KDM inhibitor)[21] (250 nL of 10 mM) was dispensed into column 24 as a no enzyme control. KDM4A (300 nM) was dispensed (25 µl) into each well of the plate and allowed to pre-incubate with compounds for 15 minutes. Reaction was initiated by dispensing of 25 µL of assay buffer containing substrate and co-factors (200 µM LAA, 20 µM FAS, 20 µM 2OG and 20 µM H3K9Me3 peptide); reactions were allowed to progress for 50 minutes, then stopped by addition of 5 µL of 10% (v/v) aqueous formic

acid. The final assay concentration of DMSO was 0.5% (v/v). Assay plates were transferred to a RapidFire RF365 high throughput sampling robot and samples aspirated under vacuum onto a C4 SPE cartridge. After an aqueous wash step (0.1% v/v formic acid in water) to remove non-volatile buffer components from the C4 SPE, peptide was eluted from the C4 SPE in an organic wash step (85% v/v aqueous acetonitrile, 0.1% formic acid) onto an Agilent 6550 accurate mass Q-TOF. One cycle of sample aspiration, loading, wash and elution takes approximately 12 seconds. Peptide charge states were monitored in the positive ESI mode with a drying gas temperature of 280°C, a drying gas flow rate of 13 L/minute, nebulizer gas pressure of 40 PSI, sheath gas temperature of 350°C, sheath gas flow rate of 12 L/min and a nozzle voltage of 1000V. Ion chromatogram data was extracted for the substrate and the product methyl marks and peak area data for extracted ion chromatograms were integrated using RapidFire Integrator software (Agilent). % conversion to the dimethyl peptide product was calculated using the equation:

% conversion = 100 x dimethyl / (dimethyl + trimethyl peptide)

IC_{50} data were determined from non-linear regression plots using GraphPad prism 6.0

MALDI-TOF MS OGFOD1 Hydroxylation Assays

Recombinant human OGFOD1 was prepared as reported, and stored at -80 °C in 50 mM HEPES (pH 7.5), 150 mM NaCl, 1 mM DTT and 5% (w/v) glycerol after purification with immobilized metal affinity chromatography.[6, 22] Activity assays were performed by determining the extent of hydroxylation of a 20-residue fragment of human RPS23 containing amino acid residues 51-70 (H_2N-VLEKVGVEAKQPNSAIRKCV-CONH_2) by matrix-assisted laser desorption/ionization time-of-flight mass spectrometry (MALDI-TOF MS) using a Waters MicromassMALDI micro MXTM mass spectrometer and MassLynxTM 4.1 as described.[23] The optimized hydroxylation assay involved incubation of OGFOD1 (1 μM) with inhibitor (1 % v/v in DMSO) in the

presence of Fe(II) (50 μM), 2OG (25 μM), L-ascorbic acid (100 μM) and $RPS23_{51-70}$ (25 μM) in HEPES (50 mM, pH 7.5) at 37 °C for 15 min. Reagent solutions were made as previously reported.[23] Reactions were quenched with formic acid (1 % v/v). Samples were prepared by mixing reaction mixture (1 μL) with α-cyano-4-hydrocinnamic acid (CHCA) solution (water:acetonitrile 1:1) (1 μL). Dose-response was assessed in 8-point triplicates. Data were analysed using GraphPad Prism 5.04.

MALDI-TOF MS vCPH Hydroxylation Assays

Recombinant viral collagen prolyl hydroxylase (vCPH) was prepared and the assay was conducted as reported .[24]

NMR Experiments

Nuclear Magnetic Resonance (NMR) spectra were recorded using a Bruker AVIII 700 MHz NMR spectrometer equipped with a 5-mm inverse cryoprobe using 3 mm MATCH NMR tubes (Cortectnet). The relaxation delay was 2 s and the $^{1}J_{CH}$ was set to 160 Hz. A 6.8 ms Q3.1000 180-degree pulse was used and selective irradiation was applied at the selective chemical shift. CODD/NODD was uniformly [^{13}C]-labelled on its proline ring. The CLIP-HSQC sequence was used for 1D HSQC experiments (without ^{13}C decoupling).[2, 25] Assay mixtures contained 50 μM 2OG, 50 μM *apo*-tPHD2, 200 μM Zn(II), 50 μM NODD/CODD and compound of interest (if necessary) buffered in 50 mM Tris-D_{11}, pH 7.5, in 90 % H_2O and 10 % D_2O. Data were processed with Bruker 3.1 software.

General Considerations For Synthesis

All reactions involving moisture-sensitive reagents were carried out under a nitrogen atmosphere using standard vacuum line techniques. Glassware was oven dried and cooled under nitrogen before use. Commercial anhydrous solvents used in reactions and HPLC grade solvents were employed for work-up and chromatography. Water was purified using an Elix UV-10 system. Aqueous solutions were made using de-ionized water. Thin layer chromatography (TLC) was carried out using Merck (Darmstadt, Germany) silica gel 60 F254 TLC plates. TLC visualization was carried out under UV light and stained with one of three stains; ninhydrin, potassium permanganate, or anisaldehyde. Chromatographic purifications were carried out using a Biotage® (Uppsala, Sweden) Isolera One or Biotage® SP4 flash purification system, using Biotage® pre-packed SNAP columns. Reactions were monitored using an Agilent (Cheshire, UK) 1200 series, 6120 quadrupole LC-MS system using a Merck Chromolith® Performance RP-18 HPLC column. Deuterated solvents were obtained from Sigma-Aldrich, and ^{1}H NMR spectra were obtained using Bruker AVANCE AVIII HD 400 nanobay (400 MHz) machine or a machine Bruker AV500 (500M Hz) with a ^{13}C cryoprobe. All signals are described in δ ppm with multiplets being denoted as singlet, doublet, triplet, quartet, and multiplet using the abbreviations s, d, t, q, and m, respectively. Chemical shifts in presented NMR spectra were referenced using residual solvent peaks with coupling constants, *J*, reported in hertz (Hz) to an accuracy of 0.5 Hz. For high-resolution mass spectrometry (HR-MS), a Bruker MicroTOF instrument with an ESI source and Time of Flight (TOF) analyzer was used. MS data are represented as a ratio of mass to charge (m/z) in Daltons. A Bruker Tensor 27 instrument was used to obtain Fourier transform infrared spectra (FT-IR). Spectroscopic grade solvents and a Perkin Elmer 241 Polarimeter were used to obtain optical rotations. All compounds were highly purified (est. >95% by ^{1}H and ^{13}C NMR).

All chemicals, reagents, and solvents were obtained from Sigma-Aldrich (Dorset, UK) and used without further purification. HPLC grade solvents were used for reactions, chromatography, and work-ups.

General Procedure for ethyl ester amide coupling

6 (1 equiv.), the relevant amine (1 equiv.) and DABACO-$(AlMe_3)_2$ (0.8 equivs) were added to a microwave vial, which was then was flushed with N_2 and evacuated (3 times) before the addition of anhydrous THF (2 ml). The reaction mixture was then heated at 130ºC for 8 minutes with microwave radiation. The reaction mixture was diluted with CH_2Cl_2 (15 ml), followed by the addition of $KNaC_4H_4O_6{\cdot}4H_2O_{aq}$ (50 ml), then stirred overnight. The phases were then separated and the organic phase was washed with water, brine and dried over Na_2SO_4. The solvent was removed in *vacuo*. The crude compound was purified by flash column chromatography using (0% - 10 % MeOH, CH_2Cl_2, 1 % NH_3) over 20 column volumes to give the desired compound.

Ethyl 4-hydroxy-2-(1H-pyrazol-1-yl) pyrimidine-5-carboxylate 6

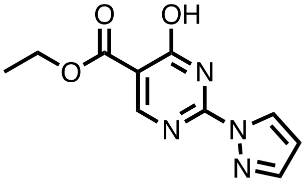

1H-Pyrazole-1-carboximide.HCl (996 mg, 6.8 mmol), diethyl ethyoxymethylmalonate (1.36 ml, 6.8 mmol) and sodium ethoxide (693 mg, 10.2 mol) were dissolved in anhydrous EtOH (10 ml). The reaction mixture was then heated with microwave radiation at 120 ºC for 90 mins. The precipitate was removed by filtration, then washed with hot EtOH and Et_2O to give **6** (960 mg, 4.10 mmol, 64%) as a cream solid. **m.p.** 157-158 ºC. **IR** ν_{max} (film) 3251, 3150, 1683, 1513 cm^{-1}. **^{1}H NMR** (400 MHz, DMSO-d_6) δ 8.46 (s, 1H, Pyrimidine (Pym)), 8.05 – 7.99 (m, 1H, Pyrazole (Pyzl)), 7.73 – 7.68 (m, 1H, Pyzl), 6.72 (m, 1H, Pyzl), 4.12 (q, J = 7 Hz, 2H, $OC\underline{H_2}CH_3$), 1.20 (t, J = 7 Hz, 3H, $OCH_2C\underline{H_3}$). **^{13}C NMR** (101 MHz, DMSO-d_6) δ 170.93, 160.80, 157.49, 152.46, 145.69,

142.79, 131.58, 129.56, 108.27, 59.77, 14.81. **HRMS** (ESI-TOF) calcd for $C_{10}H_{10}O_3N_4{}^{23}Na_1$ $[M+Na]^+$: 257.0645, found : 257.0646.

4-Hydroxy-2-(1H-pyrazol-1-yl)pyrimidine-5-carboxylic acid 7

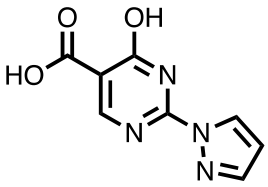

Sodium hydroxide (184 mg, 4.61 mmol) was added to a solution of **6** (500 mg, 1.53 mmol) in THF and H_2O (30ml (1:1)) and the resulting suspension stirred at room temperature for 16hr. THF was removed in *vacuo*, then the resulting mixture was washed with EtOAc (10ml), acidified to pH 2 with HCl_{aq} (1M) and extracted with EtOAc (3 x 10ml). The organic layers combined, dried over Na_2SO_4 and concentrated in *vacuo* to give **7** (391 mg, 0.189 mmol, 77 %) as a white solid.

m.p. >250 ºC. **IR** ν_{max} (film) 3310, 2770, 1730, 1570 cm^{-1}. **^{1}H NMR** (400 MHz, DMSO-d_6) δ 8.61 – 8.55 (m, 1H, Pyzl), 8.43 (s, 1H, Pym), 8.00 (s, 1H, Pyzl), 6. 69 – 6.59 (m, 1H, Pyzl). **^{13}C NMR** (101 MHz, DMSO-d_6) δ 170.92, 165.14, 158.51, 151.07, 145.62, 130.56, 122.75, 111.25. **HRMS (ESI-TOF)** calcd for $C_8H_5O_3N_4$ $[M-H]^-$: 205.0367, found 205.0363.

4-Hydroxy-*N*-(4-phenoxybenzyl)-2-(1H-pyrazol-1-yl)pyrimidine-5-carboxamide 8

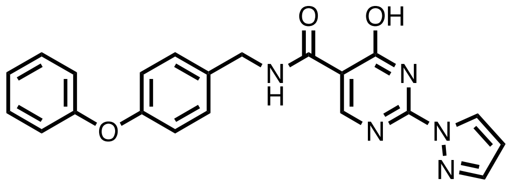

Following general procedure: **6** (100 mg, 0.425 mmol), 4-phenyoxybenzylamine (75.9 µl, 0.425 mmol) and DABCO-$(AlMe_3)_2$ (87 mg, 0.34 mmol) gave **8** (16 mg, 0.041 mmol, 10 %) as a clear oil. **IR** ν_{max} (film) 3280, 2876, 1670, 1593 cm^{-1}. **^{1}H NMR** (400 MHz, DMSO-d_6) δ 13.55 (s, 1H, OH), 8.63 (t, J=6.0 Hz, 1H, NH), 8.48 (s, 1H, Pym), 8.08 – 8.04 (m, 1H, Pyzl), 7.41 – 6.90 (m, 10H, Ar), 6.74 – 6.69 (m, 1H, Pyzl), 4.51 (d, J = 6.0 Hz, 2H,$\underline{CH_2}$). **^{13}C NMR** (101 MHz, DMSO-d_6) δ 172.38, 162.82, 157.29, 155.98, 145.33, 134.79, 130.47, 130.26, 129.68, 123.77, 119.22, 118.87, 111.13, 42.11. **HRMS** (ESI-TOF) calcd for $C_{21}H_{18}O_3N_5$ $[M+H]^+$: 388.1404, found: 388.1401.

***N*-([1,1’-Biphenyl]-4-ylmethyl)-4-hydroxy-2-(1H-pyrazol-1-yl)pyrimidine-5-carboxamide 9**

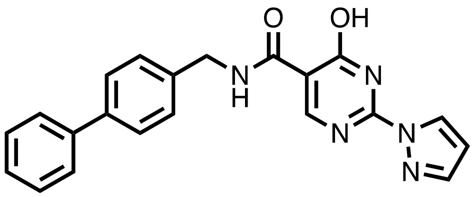

Following the general procedure, **6** (30 mg, 0.127 mmol), 4-phenylbenzylamine (23 mg , 0.120 mmol) and DABCO-$(AlMe_3)_2$ (37 mg, 0.120 mmol) gave **9** (4 mg, 0.0107 mmol, 8.5 %) as a white amorphous solid. **m.p.** >250 ℃. **IR** ν_{max} (film) 3269, 3115, 2917, 1656, 1548 cm^{-1}. **^{1}H NMR** (400 MHz, DMSO-d_6) δ 10.48 (s, 1H, OH), 8.61 – 8.48 (m, 1H, Pym), 7.85 (s, 1H, Pyzl), 7.77 – 7.26 (m, 10H, Ar), 6.56 (s, 1H, Pyzl), 4.55 (d, J = 6.0 Hz, 2H,$\underline{CH_2}$). **^{13}C NMR** (101 MHz, DMSO-d_6) δ

178.05, 165.07, 158.91, 145.55, 143.76, 142.94, 140.00, 138.77, 137.77, 134.99, 128.91, 128.91, 127.97, 127.32, 126.74, 126.61, 122.52, 103.20, 45.34. **HRMS** (ESI-TOF) calcd for $C_{21}H_{18}O_2N_5$ $[M+H]^+$: 372.1455, found: 372.1454.

N-Benzyl-4-Hydroxy-2-(1H-pyrazol-1-yl)pyrimidine-5-carboxamide 10

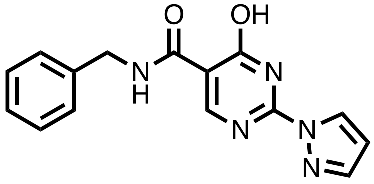

Following the general procedure, **6** (100 mg, 0.485 mmol), benzylamine (105 µl, 0.97 mmol) and DABCO-$(AlMe_3)_2$ (87 mg, 0.34 mmol) gave **10** (8 mg, 0.0271 mmol, 5.5%) as a clear oil. **IR** ν_{max} (film) 3304, 2947, 1670, 1584 cm^{-1}. **^{1}H NMR** (400 MHz, DMSO-d_6) δ 8.46 (s, 1H, Pym), 7.83 (m, 1H, Pyzl), 7.43 (m, 1H, Pyzl), 7.38 – 7.17 (m, 5H, Ph), 6.56 (m, 1H, Pyzl), 4.47 (m, 2H, $\underline{CH_2}$-NH). **^{13}C NMR** (101 MHz, DMSO-d_6) δ 174.01, 166.30, 156.65, 143.69, 140.07, 130.16, 128.91, 127.74, 127.58, 127.45, 127.28, 109.01, 44.12. **HRMS** (ESI-TOF) calcd for $C_{15}H_{14}O_2N_5$ $[M+H]^+$: 296.1142, found : 296.1140.

4-Hydroxy-2-(1H-pyrazol-1-yl)-N-(pyridin-2-ylmethyl)pyrimidine-5-carboxamide 11

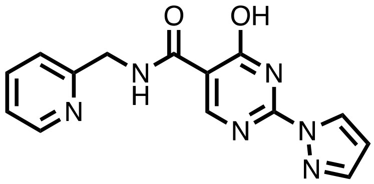

Following the general procedure, **6** (100 mg, 0.425 mmol), 2-picolylamine (43 µl, 0.425 mmol) and DABCO-$(AlMe_3)_2$ (87 mg, 0.34 mmol) gave **11** (7 mg, 0.0236 mmol, 5.5%) as a clear oil. **IR** ν_{max}

(film) 3296, 2932, 1643, 1554 cm^{-1}. **^{1}H NMR** (400 MHz, DMSO-d_6) δ 11.10 (t, J = 6 Hz, 1H, N<u>H</u>), 8.54 (s, 1H, Pym), 8.53 – 8.48 (m, 2H), 7.79 – 7.73 (m, 1H), 7.69 – 7.66 (m, 1H), 7.34 – 7.23 (m, 2H), 6.45 – 6.41 (m, 1H,Pyzl), 4.58 (d, J = 6 Hz, 2H, C<u>H$_2$</u>). **^{13}C NMR** (101 MHz, DMSO-d_6) δ 172.88, 166.63, 159.54, 158.34, 149.38, 141.91, 137.20, 129.29, 122.45, 121.52, 107.52, 44.39. **HRMS** (ESI-TOF) calcd for $C_{14}H_{13}O_2N_6$ $[M+H]^+$: 297.1095, found: 297.1092.

N-(Cyclohexylmethyl)-4-hydroxy-2-(1H-pyrazol-1-yl)pyrimidine-5-carboxamide 12

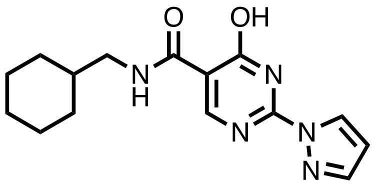

Following the general procedure, **6** (100 mg, 0.425 mmol), cyclohexane methylamine (53 µl, 0.425 mmol) and DABCO-$(AlMe_3)_2$ (87 mg, 0.34 mmol) gave **12** (22 mg, 0.073 mmol, 17.5 %) as a clear oil. **IR** ν_{max} (film) 3310, 2905, 1532 cm^{-1}. **^{1}H NMR** (400 MHz, DMSO-d_6) δ 9.68 – 9.55 (m, 1H, NH), 8.60 (s, 1H, Pym), 8.46 (m, 1H, Pyzl), 7.99 (m, 1H, Pyzl), 6.67 (m, 1H, Pyzl), 3.16 (d, J = 7.5 Hz, 2H, NH<u>CH$_2$</u>), 1.73 – 0.85 (m, 11H). **^{13}C NMR** (101 MHz, DMSO-d_6) δ 175.63, 169.27, 151.12, 144.73, 130.03, 121.26, 107.17, 45.05, 38.04, 30.87, 26.47, 25.86. **HRMS** (ESI-TOF) calcd for $C_{15}H_{20}O_2N_5$ $[M+H]^+$: 302.1612, found : 302.1615.

4-Hydroxy-2-(1H-pyrazol-1-yl)-N-(3-(trifluoromethyl)benzyl)pyrimidine-5-carboxamide 13

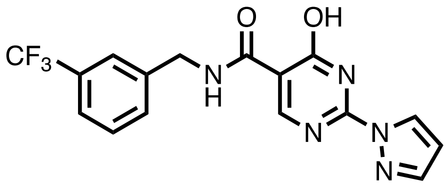

Following the general procedure, **6** (100 mg, 0.425 mmol), 3-(trifluoromethyl)benzylamine (61 µl, 0.425 mmol) and DABCO-$(AlMe_3)_2$ (87 mg, 0.34 mmol) gave **13** (34 mg, 0.093 mmol, 22%) as a white solid. **m.p.** 227-232 ºC. **IR** $_{\nu max}$ (film) 3282, 3119, 1668 cm^{-1}. **^{1}H NMR** (400 MHz, DMSO-d_6) δ 13.58 (s, 1H, OH), 9.82 (t, J = 6.0 Hz, 1H, NH), 8.48 (s, 1H, Pym), 8.10 – 8.01 (m, 1H, Pyzl), 7.74 – 7.49 (m, 5H, Ar), 6.76 – 6.68 (m, 1H, Pyzl), 4.61 (d, J = 6.0 Hz, 2H, $\underline{CH_2}$). **^{13}C NMR** (101 MHz, DMSO-d_6) δ 177.56, 169.33, 162.64, 150.40, 148.40, 148.24, 144.88, 140.89, 131.57, 129.78, 129.44, 128.91, 125.59, 123.90, 123.61, 122.89, 110.67, 41.82. **HRMS** (ESI-TOF) calcd for $C_{16}H_{12}O_2N_5F_3{}^{23}Na_1$ [M+Na]$^+$: 386.0835, found: 386.0836.

***N*-(Benzo[d][1,3]dioxol-5-ylmethyl)-4-hydroxy-2-(1H-pyrazol-1-yl)pyrimidine-5-carboxamide 14**

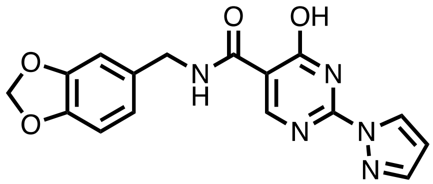

Following the general procedure, **6** (100 mg, 0.425 mmol), 3,4(methylenedioxy)-benzylamine (52 µl, 0.425 mmol) and DABCO-$(AlMe_3)_2$ (87 mg, 0.34 mmol) gave **14** (7 mg, 0.0205 mmol, 5 %) as white solid. **m.p.** >250 ºC. **IR** $_{\nu max}$ (film) 3200, 3121, 1668, 1559 cm^{-1}. **^{1}H NMR** (400 MHz, DMSO-d_6) δ 9.66 (s, 1H, OH), 8.63 (d, J = 6.0 Hz, 1H, NH), 8.46 (s, 1H, Pym), 8.06 (m, 1H, Pyzl), 6.94 – 6.68 (m, 4H, Ar), 5.98 (s, 2H, O-$\underline{CH_2}$-O), 4.42 (d, J = 6.0 Hz, 2H, $\underline{CH_2}$-NH). **^{13}C NMR** (101

MHz, DMSO-d_6) δ 176.74, 162.70, 155.36, 147.77, 146.69, 145.34, 133.42, 130.26, 121.22, 111.14, 108.60, 101.33, 42.50, 40.62, 40.41. **HRMS** (ESI-TOF) calcd for $C_{16}H_{13}O_4N_5{}^{23}Na_1$ $[M+Na]^+$: 362.0865, found: 362.0861.

N-([1,1'-Biphenyl]-4-yl)-4-hydroxy-2-(1H-pyrazol-1-yl)pyrimidine-5-carboxamide 15

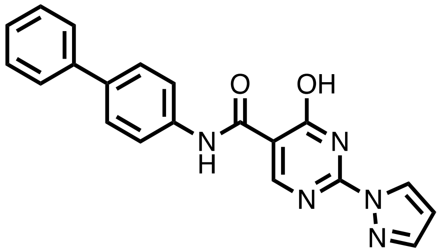

Following the general procedure, **6** (100 mg, 0.425 mmol), 4-aminobiphenyl (77 mg, 0.425 mmol) and DABCO-$(AlMe_3)_2$ (87 mg, 0.34 mmol) gave **15** (6 mg, 0.016 mmol, 4 %) as a white solid. **m.p.** >250 ºC . **IR** ν_{max} (film) 3283, 3087, 1670, 1543 cm^{-1}. **^{1}H NMR** (400 MHz, DMSO-d_6) δ 13.20 (s, 1H, OH), 8.70 (s, 1H, NH), 8.54 (s, 1H, Pym), 7.84 – 7.28 (m, 11H, Ar), 6.50 (m, 1H, Pyzl). **^{13}C NMR** (101 MHz, DMSO-d_6) δ 176.79, 164.62, 158.73, 157.19, 146.81, 142.49, 140.26, 139.31, 134.89, 129.51, 129.36, 127.56, 127.38, 126.64, 120.17, 108.10. **HRMS** (ESI-TOF) calcd for $C_{20}H_{14}O_2N_5$ $[M-H]^-$: 356.1153, found: 356.1155.

(*R*)-4-Hydroxy-N-(2-hydroxy-1-phenylethyl)-2-(1H-pyrazol-1-yl)pyrimidine-5-carboxamide 16

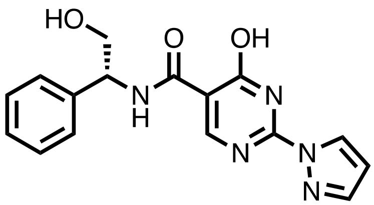

Following the general procedure, **6** (60 mg, 0.291 mmol), (R)-(-)-2-Phenylglycinol (119 mg, 0.873 mmol) and DABCO-$(AlMe_3)_2$ (60 mg, 0.232 mmol) gave **16** (11 mg, 0.033 mmol, 11 %) as a clear oil. **IR** ν_{max} (film) 3383, 1638, 1397 cm^{-1}. **^{1}H NMR** (400 MHz, DMSO-d_6) δ 11.03 (s, 1H, OH-Pym), 8.73 – 8.27 (m, 2H), 7.72 (s, 1H, Pyzl), 7.55 – 7.03 (m, 6H, Ar), 6.52 – 6.41 (m, 1H, Pyzl), 5.05 (dd, J = 8.0, 5.5 Hz, 1H, C<u>H</u>), 3.83 – 3.26 (m, 2H, CH_2). **^{13}C NMR** (101 MHz, DMSO-d_6) δ 173.73, 167.24, 157.90, 146.10, 142.28, 139.31, 129.39, 128.55, 127.40, 127.09, 120.81, 107.92, 65.55, 54.84. **HRMS** (ESI-TOF) calcd for $C_{16}H_{16}O_3N_5$ $[M+H]^+$: 326.1248, found : 326.1248.

(*S*)-4-Hydroxy-N-(2-hydroxy-1-phenylethyl)-2-(1H-pyrazol-1-yl)pyrimidine-5-carboxamide 17

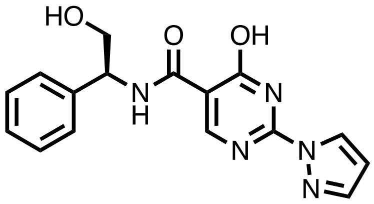

Following the general procedure, **6** (100 mg, 0.425 mmol), (S)-(-)-2-phenylglycinol (58 mg, 0.425 mmol) and DABCO-$(AlMe_3)_2$ (87 mg, 0.34 mmol) gave **17** (14 mg, 0.043 mmol, 10 %) as a clear oil. **IR** ν_{max} (film) 3412, 1643, 1547 cm^{-1}. **^{1}H NMR** (400 MHz, DMSO-d_6) δ 11.09 (t, J = 8.0 Hz, 1H, OH-CH_2), 8.56 – 8.49 (s, 1H, Pym), 7.74 – 7.66 (m, 1H, Pyzl), 7.40 – 7.20 (m, 6H, Ar), 6.46 (s, 1H, Pyzl), 5.05 (dd, J = 8.0, 5.5 Hz, 1H, C<u>H</u>-NH), 3.98 – 3.45 (m, 2H, C<u>H$_2$</u>-OH). **^{13}C NMR** (101 MHz, DMSO-d_6) δ 177.49, 166.42, 155.61, 143.19, 143.17, 139.06, 130.29, 129.95, 128.48, 127.46, 121.14, 106.16, 67.59, 57.62. **HRMS** (ESI-TOF) calcd or $C_{16}H_{14}O_3N_5$ $[M-H]^-$: 324.1102, found: 324. 1102.

References

[1] J. P. Holt-Martyn, A. Tumber, M. Z. Rahman, K. Lippl, W. Figg, Jr., M. A. McDonough, R. Chowdhury, C. J. Schofield, *Medchemcomm* **2019**, *10*, 500-504.

[2] T. L. Yeh, T. M. Leissing, M. I. Abboud, C. C. Thinnes, O. Atasoylu, J. P. Holt-Martyn, D. Zhang, A. Tumber, K. Lippl, C. T. Lohans, I. K. H. Leung, H. Morcrette, I. J. Clifton, T. D. W. Claridge, A. Kawamura, E. Flashman, X. Lu, P. J. Ratcliffe, R. Chowdhury, C. W. Pugh, C. J. Schofield, *Chem Sci* **2017**, *8*, 7651-7668.

[3] M. C. Chan, J. P. Holt-Martyn, C. J. Schofield, P. J. Ratcliffe, *Mol Aspects Med* **2016**, *47-48*, 54-75.

[4] R. Chowdhury, I. K. Leung, Y. M. Tian, M. I. Abboud, W. Ge, C. Domene, F. X. Cantrelle, I. Landrieu, A. P. Hardy, C. W. Pugh, P. J. Ratcliffe, T. D. Claridge, C. J. Schofield, *Nat Commun* **2016**, *7*, 12673.

[5] M. D. Rosen, H. Venkatesan, H. M. Peltier, S. D. Bembenek, K. C. Kanelakis, L. X. Zhao, B. E. Leonard, F. M. Hocutt, X. Wu, H. L. Palomino, T. I. Brondstetter, P. V. Haugh, L. Cagnon, W. Yan, L. A. Liotta, A. Young, T. Mirzadegan, N. P. Shankley, T. D. Barrett, M. H. Rabinowitz, *ACS Med Chem Lett* **2010**, *1*, 526-529.

[6] S. Horita, J. S. Scotti, C. Thinnes, Y. S. Mottaghi-Taromsari, A. Thalhammer, W. Ge, W. Aik, C. Loenarz, C. J. Schofield, M. A. McDonough, *Structure* **2015**, *23*, 639-652.

[7] J. E. Longbotham, C. Levy, L. O. Johannissen, H. Tarhonskaya, S. Jiang, C. Loenarz, E. Flashman, S. Hay, C. J. Schofield, N. S. Scrutton, *Biochemistry* **2015**, *54*, 6093-6105.

[8] L. A. McNeill, E. Flashman, M. R. Buck, K. S. Hewitson, I. J. Clifton, G. Jeschke, T. D. Claridge, D. Ehrismann, N. J. Oldham, C. J. Schofield, *Molecular bioSystems* **2005**, *1*, 321-324.

[9] M. A. McDonough, V. Li, E. Flashman, R. Chowdhury, C. Mohr, B. M. Lienard, J. Zondlo, N. J. Oldham, I. J. Clifton, J. Lewis, L. A. McNeill, R. J. Kurzeja, K. S. Hewitson, E. Yang, S. Jordan, R. S. Syed, C. J. Schofield, *Proc Natl Acad Sci U S A* **2006**, *103*, 9814-9819.

[10] G. Winter, D. G. Waterman, J. M. Parkhurst, A. S. Brewster, R. J. Gildea, M. Gerstel, L. Fuentes-Montero, M. Vollmar, T. Michels-Clark, I. D. Young, N. K. Sauter, G. Evans, *Acta Crystallogr D Struct Biol* **2018**, *74*, 85-97.

[11] M. D. Winn, C. C. Ballard, K. D. Cowtan, E. J. Dodson, P. Emsley, P. R. Evans, R. M. Keegan, E. B. Krissinel, A. G. W. Leslie, A. McCoy, S. J. McNicholas, G. N. Murshudov, N. S. Pannu, E. A. Potterton, H. R. Powell, R. J. Read, A. Vagin, K. S. Wilson, *Acta Crystallogr D Struct Biol* **2011**, *67*, 235-242.

[12] M. C. Chan, O. Atasoylu, E. Hodson, A. Tumber, I. K. Leung, R. Chowdhury, V. Gomez-Perez, M. Demetriades, A. M. Rydzik, J. Holt-Martyn, Y. M. Tian, T. Bishop, T. D. Claridge, A. Kawamura, C. W. Pugh, P. J. Ratcliffe, C. J. Schofield, *PLoS One* **2015**, *10*, e0132004.

[13] A. J. McCoy, R. W. Grosse-Kunstleve, P. D. Adams, M. D. Winn, L. C. Storoni, R. J. Read, *J Appl Crystallogr* **2007**, *40*, 658-674.

[14] P. D. Adams, P. V. Afonine, G. Bunkoczi, V. B. Chen, I. W. Davis, N. Echols, J. J. Headd, L.-W. Hung, G. J. Kapral, R. W. Grosse-Kunstleve, A. J. McCoy, N. W. Moriarty, R. Oeffner, R. J. Read, D. C. Richardson, J. S. Richardson, T. C. Terwilliger, P. H. Zwart, *Acta Crystallogr D Struct Biol* **2010**, *66*, 213-221.

[15] P. Emsley, K. Cowtan, *Acta Crystallogr D Struct Biol* **2004**, *60*, 2126-2132.

[16] A. P. Hardy, I. Prokes, L. Kelly, I. D. Campbell, C. J. Schofield, *J Mol Biol* **2009**, *392*, 994-1006.

[17] L. Kelly, M. A. McDonough, M. L. Coleman, P. J. Ratcliffe, C. J. Schofield, *Molecular bioSystems* **2009**, *5*, 52-58.

[18] aJ. M. Elkins, K. S. Hewitson, L. A. McNeill, J. F. Seibel, I. Schlemminger, C. W. Pugh, P. J. Ratcliffe, C. J. Schofield, *J Biol Chem* **2003**, *278*, 1802-1806; bK. S. Hewitson, L. A. McNeill, M. V. Riordan, Y. M. Tian, A. N. Bullock, R. W. Welford, J. M. Elkins, N. J.

Oldham, S. Bhattacharya, J. M. Gleadle, P. J. Ratcliffe, C. W. Pugh, C. J. Schofield, *J Biol Chem* **2002**, *277*, 26351-26355.
[19] R. L. Hancock, M. I. Abboud, T. J. Smart, E. Flashman, A. Kawamura, C. J. Schofield, R. J. Hopkinson, *ChemBioChem* **2018**, *19*, 917-921.
[20] S. E. Hutchinson, M. V. Leveridge, M. L. Heathcote, P. Francis, L. Williams, M. Gee, J. Munoz-Muriedas, B. Leavens, A. Shillings, E. Jones, P. Homes, S. Baddeley, C. W. Chung, A. Bridges, A. Argyrou, *J Biomol Screen* **2012**, *17*, 39-48.
[21] N. R. Rose, S. S. Ng, J. Mecinovic, B. M. Lienard, S. H. Bello, Z. Sun, M. A. McDonough, U. Oppermann, C. J. Schofield, *J Med Chem* **2008**, *51*, 7053-7056.
[22] R. S. Singleton, P. Liu-Yi, F. Formenti, W. Ge, R. Sekirnik, R. Fischer, J. Adam, P. J. Pollard, A. Wolf, A. Thalhammer, C. Loenarz, E. Flashman, A. Yamamoto, M. L. Coleman, B. M. Kessler, P. Wappner, C. J. Schofield, P. J. Ratcliffe, M. E. Cockman, *Proc Natl Acad Sci U S A* **2014**, *111*, 4031-4036.
[23] R. Chowdhury, K. K. Yeoh, Y. M. Tian, L. Hillringhaus, E. A. Bagg, N. R. Rose, I. K. Leung, X. S. Li, E. C. Woon, M. Yang, M. A. McDonough, O. N. King, I. J. Clifton, R. J. Klose, T. D. Claridge, P. J. Ratcliffe, C. J. Schofield, A. Kawamura, *EMBO Rep* **2011**, *12*, 463-469.
[24] G. W. Langley, M. I. Abboud, C. T. Lohans, C. J. Schofield, *Bioorg Med Chem* **2019**, *27*, 2405-2412.
[25] M. I. Abboud, T. E. McAllister, I. K. H. Leung, R. Chowdhury, C. Jorgensen, C. Domene, J. Mecinovic, K. Lippl, R. L. Hancock, R. J. Hopkinson, A. Kawamura, T. D. W. Claridge, C. J. Schofield, *Chem Commun* **2018**, *54*, 3130-3133.
